# Transparent SiON/Ag/SiON multilayer passivation grown on a flexible polyethersulfone substrate using a continuous roll-to-roll sputtering system

**DOI:** 10.1186/1556-276X-7-69

**Published:** 2012-01-05

**Authors:** Han-Ki Kim, Chung-Ki Cho

**Affiliations:** 1 Department of Advanced Materials Engineering for Information and Electronics, Kyung Hee University, 1 Seocheon-dong, Yongin-si, Gyeonggi-do, 446-701, South Korea

## Abstract

We have investigated the characteristics of a silicon oxynitride/silver/silicon oxynitride [SiON/Ag/SiON] multilayer passivation grown using a specially designed roll-to-roll [R2R] sputtering system on a flexible polyethersulfone substrate. Optical, structural, and surface properties of the R2R grown SiON/Ag/SiON multilayer were investigated as a function of the SiON thickness at a constant Ag thickness of 12 nm. The flexible SiON/Ag/SiON multilayer has a high optical transmittance of 87.7% at optimized conditions due to the antireflection and surface plasmon effects in the oxide-metal-oxide structure. The water vapor transmission rate of the SiON/Ag/SiON multilayer is 0.031 g/m^2 ^day at an optimized SiON thickness of 110 nm. This indicates that R2R grown SiON/Ag/SiON is a promising thin-film passivation for flexible organic light-emitting diodes and flexible organic photovoltaics due to its simple and low-temperature process.

## Introduction

Rapid progress in organic-based flexible optoelectronics such as flexible organic light-emitting diodes [OLEDs] and organic photovoltaics [OPVs] required a high-performance thin-film passivation because both lifetime and performance of the flexible OLEDs and OPVs are critically affected by the quality of the encapsulation [1-3]. The long-term stability of flexible OLEDs and OPVs is still limited due to the instability of the luminescent organic materials and low work function metals, interfacial reactions, and chemical reactions of the organic layers with oxygen and moisture in air [4]. For those reasons, several types of encapsulation techniques have been extensively explored to improve the long-term stability of flexible OLEDs or OPVs. In particular, thin-film passivation has been considered as the most desirable encapsulation for flexible OLEDs and OPVs due to its simplicity, thinness, and flexibility. Although various SiN_x_, SiO_x_, SiO_x_N_y_, AlO_x_, and Al_2_O_3_:N films have been reported, a single-layer-based thin-film passivation is not sufficiently dense to protect flexible optoelectronic devices from permeation by moisture and oxygen [5-9]. Therefore, multilayer passivation, such as Barix coating or NONON (SiN_x_/SiO_2_/SiN_x_/SiO_2_/SiN_x_) structures, has been proposed as a means to achieve ultra high barrier properties for flexible OLEDs or OPVs [10,11]. However, Barix coating or the NONON structure still has not been employed in mass production of OLEDs due to its complicated process and long process time. We also reported that Al_2_O_3_/Ag/Al_2_O_3 _multilayer thin-film passivation has a high transmittance of 86.44% and a low water vapor transmission rate [WVTR] due to the SPR effects of the Ag interlayer and the effective multilayer structure that prevent the intrusion of water vapor [12]. In a multilayer barrier, control of the Ag thickness is very important because the antireflection effect for high transparency is critically dependent on the thickness and morphology of the inserted Ag layer. However, a roll-to-roll [R2R] sputter-grown silicon oxynitride/silver/silicon oxynitride [SiON/Ag/SiON] multilayer has not been investigated for thin-film passivation even though it has various advantages such as high transparency and possibility of a simple R2R process.

In this work, we report on the characteristics of SiON/Ag/SiON multilayer passivation grown on a flexible polyethersulfone [PES] substrate using a specially designed R2R sputtering system. Optical, structural, and surface properties of the R2R-grown SiON/Ag/SiON multilayer were investigated as a function of the SiON thickness. Despite the low process temperature used, a SiON/Ag/SiON multilayer passivation showed a low WVTR of 0.031 g/m^2 ^day and a high transmittance of 87.7% at an optimized SiON thickness of 110 nm.

### Experimental detail

**Figure 1:**
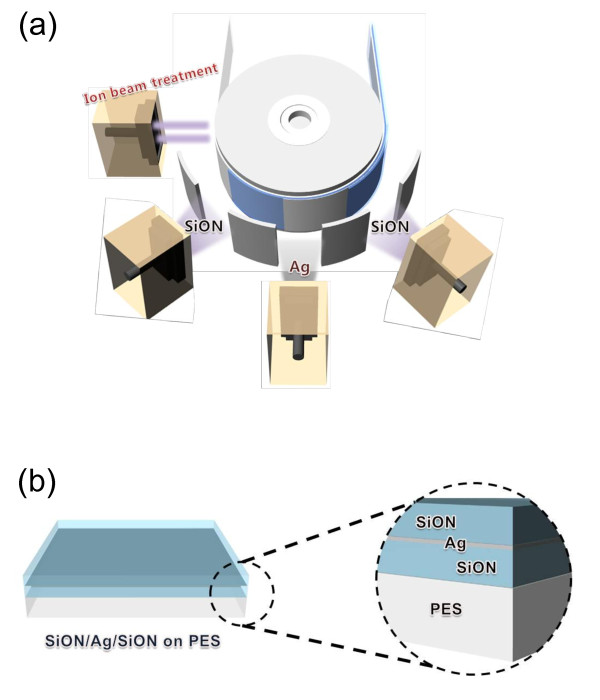
**Schematic and structure**. (**a**) Schematic of a continuous R2R sputtering process and (**b**) structure of the SiON/Ag/SiON multilayer passivation on PES substrate.

The flexible SiON/Ag/SiON multilayer was sputtered on a flexible PES substrate as a function of the SiON thickness using a continuous R2R sputtering system as shown in Figure 1a[13]. The SiON ceramic and Ag metal targets were placed at a distance of 100 mm from the PES substrate, mechanically contacted on the cooling drum. Before the sputtering of the bottom SiON layer, a flexible PES substrate was pretreated with Ar ion beam treatment at a DC-pulsed power of 100 W to enhance adhesion between the PES substrate and the bottom SiON layer. After the ion beam treatment, the bottom SiON layer was sputtered on the PES substrate at a constant base pressure of 1.0 × 10^-6 ^Torr, a working pressure of 3 mTorr, an Ar/O_2 _flow rate of 30/2 sccm, and a rolling speed of 0.1 cm/s as a function of the SiON target RF power. Subsequently, a constant Ag layer was sputtered on the bottom SiON layer using a DC power of 350 W. The top SiON layer was sputtered on the Ag layer with identical sputtering conditions used for the bottom SiON layer. As shown in Figure 1a, the SiON/Ag/SiON multilayer was continuously deposited without breaking the vacuum in the R2R sputter system. Figure 1b showed the schematic structure of the SiON/Ag/SiON multilayer sputtered on the PES substrate. The thickness of the SiON/Ag/SiON multilayer was measured by a surface profilometer. The optical transmittance of the SiON/Ag/SiON multilayer was measured in a wavelength range from 300 to 1100 nm using a UV/Visible spectrometer as a function of the SiON thickness. In addition, the surface morphology of the top SiON layer in the SiON/Ag/SiON multilayer was investigated by a field emission scanning electron microscope [FESEM]. Moreover, the structural properties of the SiON/Ag/SiON multilayer were examined by X-ray diffraction [XRD] and high resolution transmission electron microscope [HRTEM]. Furthermore, the WVTR value for the SiON/Ag/SiON multilayer passivation grown on the flexible PES substrate (50 mm × 50 mm) was measured by a MOCON tester (PERMATRAN-W Model 3/33, MOCON Inc., Minneapolis, MN, USA) for 20 h. The calibration was conducted using a standard sample supported by MOCON under a flow of 10 sccm water vapor at 37.8°C.

## Results and discussion

**Figure 2:**
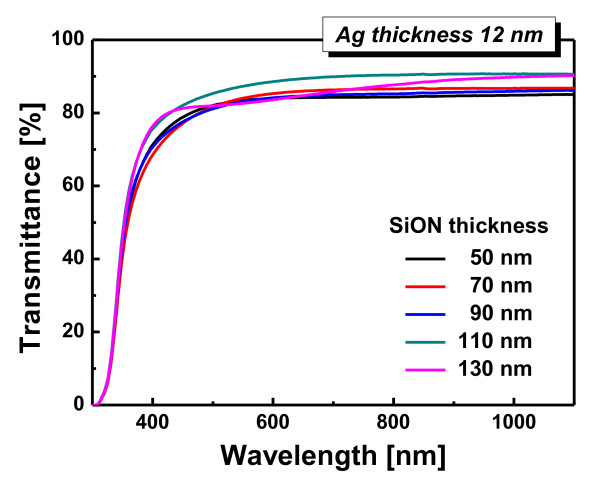
**Optical properties**. Optical transmittance of the R2R-grown SiON/Ag/SiON (Ag 12 nm) multilayer sputtered on a PES substrate as a function of the top and bottom SiON thickness (50 nm to 130 nm).

Figure 2 shows the optical transmittance of the SiON/Ag/SiON multilayer, with an Ag thickness of 12 nm, grown on the flexible PES substrate as a function of the top and bottom SiON thicknesses of layers from 50 to 130 nm at a constant Ag thickness. It was found that the optical transmittance of the SiON/Ag/SiON multilayer electrode was dependent on the thickness of the SiON layer. The SiON/Ag/SiON multilayers with SiON thicknesses of 50 to 130 nm show a similar optical transmittance. However, the SiON/Ag/SiON multilayer with a SiON thickness of 110 nm shows an abrupt increase in optical transmittance up to 87.7% at a 550-nm wavelength region due to the antireflection and surface plasmon resonance effects caused by the oxide-metal-oxide multilayer structure [12,14]. However, a further increase in the SiON thickness (130 nm) leads to the decrease of transmittance.

**Figure 3:**
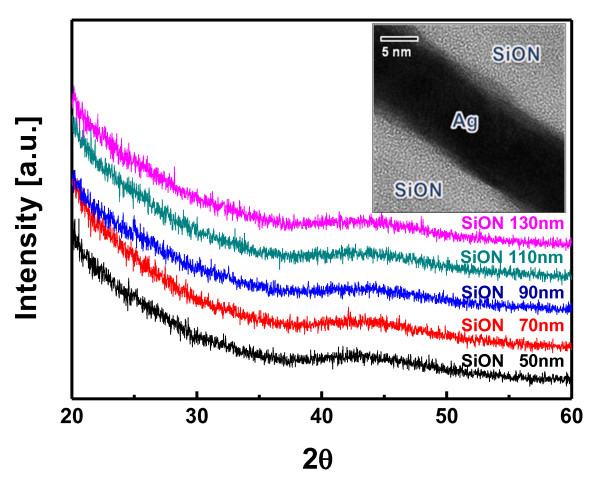
**X-ray diffraction analysis and HRTEM image**. XRD plots of the R2R-grown SiON/Ag/SiON (Ag 12 nm) multilayer sputtered on a PES substrate as a function of the top and bottom SiON thickness. The inset shows the cross-sectional HRTEM image of the SiON (110 nm)/Ag (12 nm)/SiON (110 nm) multilayer.

Figure 3 shows the XRD results of SiON/Ag/SiON electrodes as a function of the SiON thickness, with an inset of the cross-sectional HRTEM image of the optimized SiON/Ag/SiON multilayer. All XRD plots of the flexible SiON/Ag/SiON multilayer show only broad peaks at regions 40° to 50° regardless of the SiON thickness, which is indicative of an amorphous structure of SiON layer. Due to the resolution limit of our XRD system, the Ag (12 nm) peak was not detected. Because the PES substrate temperature is effectively kept low during the continuous sputtering process by the cooling drum, all the SiON layers show amorphous structures. As a barrier layer, the amorphous structure is beneficial because there are no paths for intrusion of humidity and oxygen gas. In addition, mechanical properties of the amorphous structure are more robust than that of the crystalline structure when it bent. The cross-sectional images clearly demonstrate well-defined bottom SiON, Ag, and top SiON layers without interface layers. These sharp interfaces indicate that there were no interface reactions and no formation of interfacial oxide layers between the SiON and Ag layers. Moreover, the uniform contrast of the SiON layers indicates that the structures of SiON were completely amorphous as expected from XRD results. However, the inserted Ag layer existed in crystalline form which is inconstant with the XRD results. Although the Ag peak did not appear in the XRD plot due to resolution limitation, the Ag layer had a crystalline structure as reported previously in the OMO structure [12-14].

**Figure 4:**
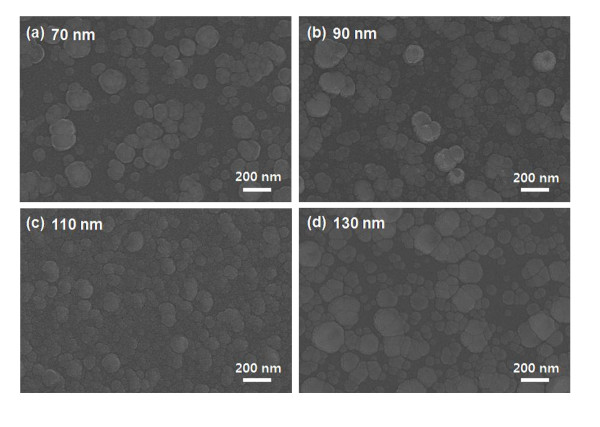
**FESEM images**. Surface FESEM images of the top SiON layer in the SiON/Ag/SiON multilayer as a function of the SiON thickness. Top SiON layer surface with thicknesses of (**a**) 70, (**b**) 90, (**c**) 110, and (**d**) 130 nm, respectively.

Figure 4 shows the surface morphology of the top SiON layer as a function of its thickness at a constant Ag thickness of 12 nm. The R2R sputter-grown SiON top layer showed a fairly rough surface morphology. An increase of the SiON thickness from 70 to 130 nm resulted in a rough surface of the top SiON layer. The rough surface of the top SiON layer could be attributed to the high kinetic energy of sputtered SiON particles and reaction with nitrogen of the SiON film with oxygen ambient during the sputtering process. All samples showed an island-like agglomeration on the surface of the top SiON layer.

**Figure 5:**
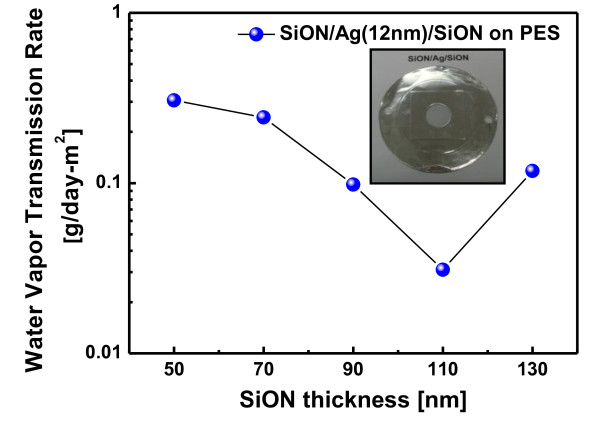
**WVTR values**. WVTR values of SiON/Ag/SiON multilayer passivation as a function of the SiON thickness.

Figure 5 shows the WVTR value of the SiON/Ag/SiON multilayer passivation grown on the flexible PES substrate as a function of both top and bottom SiON thicknesses. Due to the small size of the SiON/Ag/SiON multilayer samples, the WVTR values for all multilayer passivation were measured by packaging the samples as it is shown in the inset picture. As shown in Figure 5, the WVTR value of the SiON/Ag/SiON multilayer depends on the thickness of the SiON layers. As the SiON thickness in the SiON/Ag/SiON multilayer increases, the WVTR value monotonically decreases because both SiON layers can effectively prevent the intrusion of water vapor. Compared with the WVTR value (0.306 g/m^2 ^day) of the SiON thickness of 50 nm, the WVTR value (0.031 g/m^2 ^day) of the SiON/Ag/SiON multilayer with a SiON thickness of 110 nm is much lower. However, a further increase of the SiON thickness leads to an increase of the WVTR value. The increase in WVTR value of the SiON/Ag/SiON with the 130-nm-thick SiON layer could be attributed to the rough surface as shown in Figure 4d. This rough surface with island-like agglomerated subgrains could lead to the formation of a diffusion path for oxygen atoms or moisture from the surface to the substrate. This rough surface is caused by a chemical reaction of the SiON and oxygen ambient. Due to different bond enthalpies of Si-O (799.6 kJ/mol), O-O (498.4 kJ/mol), N-N (945.3 kJ/mol), and Si-N (470 kJ/mol), the formation of Si-O and N-N bonds are energetically favorable during sputtering of the SiON target in an oxygen ambient. Therefore, the presence of an oxygen ambient leads to the ejection of diatomic nitrogen into the ambient from the SiON target, and this resulted in a SiO_x _film with a very rough surface morphology. Considering the optical transparency and WVTR value, we decided the optimized thickness value of the top and bottom SiON layers as 110 nm. Compared to a previously reported WVTR value of multilayer thin-film passivation [12], the SiON/Ag/SiON multilayer passivation showed a higher value due to the rough surface morphology of the SiON layers as shown in Figure 4. Therefore, we believe that further optimization of the top SiON morphology and density could improve the performance of the SiON/Ag/SiON multilayer passivation.

## Conclusions

SiON/Ag/SiON multilayer passivation prepared by continuous R2R sputtering was investigated as a function of the top and bottom SiON thickness. The SiON/Ag/SiON multilayer thin-film passivation on the PES substrate has a high transmittance of 87.7% and a low WVTR due to the antireflection and surface plasmon effects of the Ag interlayer and the effective multilayer structure that prevent the intrusion of water vapor. At a SiON thickness of 110 nm, the R2R-grown SiON/Ag/SiON multilayer showed a WVTR value of 0.031 g/m^2 ^day. These findings indicate that R2R-grown SiON/Ag/SiON is a promising thin-film passivation for flexible OLEDs and OPVs due to its simple and low-temperature process.

## Competing interests

The authors declare that they have no competing interests.

## Authors' contributions

CKC carried out the R2R sputtering process and analysis of the SiON/Ag/SiON multilayer passivation layer. HKK designed the experiments and wrote the manuscript. All authors read and approved the final manuscript.
